# Age‐Dependent Differences in Cerebellar CB1 Receptor Expression and Its Association With Impulsivity and Alcohol Intake in Rats

**DOI:** 10.1111/adb.70107

**Published:** 2025-11-29

**Authors:** Johana P. Gómez‐Villatoro, Yalitza A. Alvarado‐Ramirez, Octavio Amancio‐Belmont, Mónica Méndez‐Díaz

**Affiliations:** ^1^ Laboratorio de Ontogenia y Adicciones, Departamento de Fisiología, Facultad de Medicina Universidad Nacional Autónoma de México Mexico City Mexico; ^2^ División de Neurociencias, Instituto de Fisiología Celular Universidad Nacional Autónoma de México Mexico City Mexico

**Keywords:** adolescence, aged, alcohol use disorder, cannabinoid one receptor, cerebellum, impulsivity

## Abstract

The cannabinoid receptor 1 (CB1R) in the hippocampus has been involved in impulsivity and alcohol consumption in adolescent rats. However, the role of CB1R in the cerebellum, despite that structure's exceptionally high CB1R density, remains unexplored. We therefore tested the hypothesis that cerebellar CB1R contributes to regulating impulsive behaviour, alcohol consumption and seeking. Male Wistar rats, adolescent (PND25), adult (PND90) and aged (PND365), were assessed on the delay discounting task (DDT) and for voluntary alcohol intake‐conditioned place preference (ACPP). CB1R levels were quantified via immunofluorescence in the CRUS II and interposed nucleus. Separate cohorts were used for voluntary alcohol consumption and alcohol‐conditioned place preference (ACPP). Results showed that adolescent rats displayed significantly greater impulsivity and alcohol consumption than adults and aged rats, and only adolescents developed ACPP. CB1R expression peaked in adolescents across both cerebellar regions, but only Crus II levels correlated positively with impulsivity (*k* value), indicating a region‐ and age‐specific contribution. These data reveal, for the first time, that CB1R in cerebellar Crus II selectively underpins adolescent impulsive choice and alcohol consumption and seeking, highlighting both a novel brain locus and a critical developmental window for endocannabinoid modulation.

## Graphical abstract

Adolescent rats exhibited significantly higher levels of impulsivity compared to their adult and aged counterparts, and only adolescents developed alcohol‐conditioned place preference. CB1R expression in Crus II was notably higher in adolescents, which correlated with major impulsive behaviour. Together, these results emphasize the cerebellum's essential role in behavioural regulation.

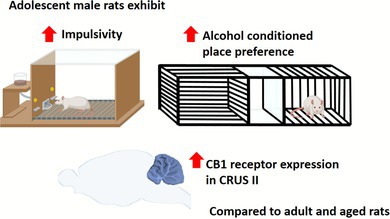

## 1 Introduction

Impulsivity is a complex, multidimensional construct characterized by two key components: impulsive action, defined as the incapacity to inhibit inappropriate responses despite potential negative outcomes, and impulsive choice, referring to deficits in delaying gratification or use of self‐control [1, 2]. Although rapid decision‐making can be adaptive in some contexts, it is often maladaptive in everyday life [3]. High impulsivity is a risk factor for substance use disorders (SUDs). Longitudinal data from young adults indicate that impulsivity predicts subsequent alcohol and drug use [4]. A positive correlation between impulsivity and the frequency of alcohol and tobacco use has been reported in adolescents [5, 6].

The cortico‐striatal network is central to the top‐down regulation of self‐control, and impairments in this network have been associated with impulsivity [7]. Besides impulsivity, adolescence is characterized by ongoing maturation of the prefrontal cortex (PFC), essential for executive control [8, 9]. Although the cerebellum expresses exceptionally high levels of CB1R, developmental trajectories of cerebellar CB1R remain poorly characterized. Anatomo‐functional studies have demonstrated that the cerebellum is connected to the PFC and subcortical regions involved in cognitive and emotional regulation, including via disynaptic pathways through thalamic nuclei [10, 11]. Functional neuroimaging studies in humans also support the involvement of cerebellar posterior regions—particularly Crus I/II—in networks associated with executive control and decision‐making [12, 13]. Emerging evidence suggests that the cerebellum may contribute to the modulation of self‐control regulatory functions, including impulsive behaviour, through its influence on prefrontal circuits.

Cerebellar abnormalities have been linked to impulsive traits. Cerebellar ataxia patients show elevated scores on the Barratt Impulsiveness Scale (BIS‐11) [14]. Impulsivity has also been reported after strokes involving cerebellar lobules VI and CRUS I [15]. In nonclinical populations, higher impulsivity correlates with increased grey matter volume in the cerebellar vermis and reduced volume in the right posterior cerebellum [8], supporting the cerebellum's role in self‐control.

The cerebellum has also been implicated in SUDs. Neuroimaging studies found reduced grey and white matter volumes in lobules VI, CRUS I and CRUS II, in cocaine or alcohol dependence subjects [16, 17]. Altered cerebellar–striatal connectivity has been observed in heavy smokers [18], reinforcing that cerebellar dysfunction may contribute to SUDs. However, it remains unclear whether these cerebellar alterations precede substance use or result from it, an issue readily addressed using controlled animal models.

The cerebellum expresses high levels of cannabinoid receptor type 1 (CB1R). Although previous research has shown age‐related differences in CB1R expression in cortical and limbic structures [19, 20], developmental trajectories of cerebellar CB1R remain poorly characterized.

CB1R activation enhances drug‐seeking behaviour and impulsivity. Early‐life stressors have been linked to increased CB1R expression in the nucleus accumbens, facilitating alcohol intake later in life [21]. CB1R has also been implicated in impulsivity; transgenic rats (F238L) overexpressing CB1R in adulthood exhibit high‐risk/novelty‐seeking behaviour, increased social interaction, impulsivity and heightened sensitivity to rewards from cocaine and palatable foods [22]. Furthermore, adolescent rats show both elevated CB1R expression in CA3 and greater vulnerability to alcohol‐seeking compared to adults [20].

Here, we investigated associations between cerebellar CB1R expression and both impulsive choice and alcohol‐seeking across adolescent, adult and aged male rats. Using the delay discounting task and alcohol‐conditioned place preference, we compared adolescent, adult and aged male rats. We hypothesized that adolescent rats would display higher impulsivity and greater alcohol intake, paralleled by increased CB1R expression in cerebellar regions involved in cognition (CRUS II) and motor integration (interposed nucleus). By including aged rats, we sought to explore whether reduced impulsivity and alcohol consumption in older individuals are associated with decreased CB1R expression, providing a lifespan perspective on the cerebellar endocannabinoid system and its relevance to behavioural control.

## 2 Material and Methods

Fifty‐nine male Wistar rats (20 PND25, 20 PND90 and 19 PND365) were obtained from the UNAM Faculty of Medicine Bioterium and housed in groups of three in acrylic cages (43 × 53 × 20 cm) under standard conditions (22°C ± 1°C; 52% humidity; 12:12 h light/dark, lights off at 08:00), with ad libitum access to water and Purina Lab Chow. All procedures complied with NOM‐062‐ZOO‐1999.

**FIGURE 1:**
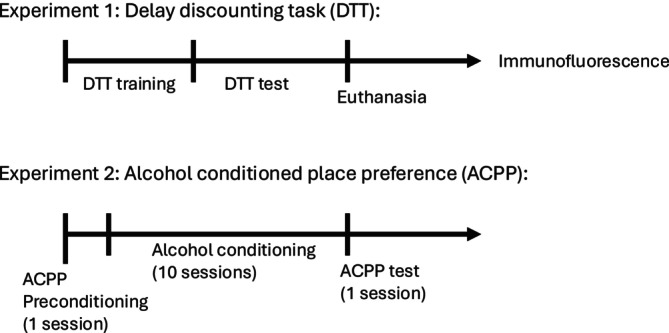
Experimental timeline for delay discounting task (DTT), alcohol conditioned place preference (ACPP) and immunofluorescence procedures.

Separate groups of animals were used for each behavioural task to avoid carryover or confounding effects. Rats subjected to the CPP procedure and alcohol exposure were exclusively used for that protocol. Conversely, animals assigned to the delay discounting task were not exposed to alcohol and were subsequently used for brain collection and immunofluorescence analysis (Figure 1).

### 2.1 Delay Discounting Task (DDT)

This task assesses the extent to which subjects prefer smaller, immediate rewards over larger, delayed ones. A higher preference for immediate rewards is interpreted as greater impulsive choice or delay discounting. This behavioural pattern reflects a core component of impulsivity, commonly associated with impaired self‐control and increased vulnerability to substance use, as it indicates a diminished ability to tolerate delays in gratification in favour of more advantageous outcomes [23].

Before training, rats were food restricted until they reached 80% of their body weight, as previously described [19, 20].

#### 2.1.1 Apparatus

Operant chambers (MED Associates Inc., Model ENV‐007CT) housed within a sound‐attenuating cubicle (Model ENV‐018V) were used. Chambers contained a metal food receptacle (ENV‐200R2M), a pellet dispenser (Model ENV‐203 M‐45), two cue lights (ENV‐215M) and two retractable levers (Model ENV‐112CM).

#### 2.1.2 Training

Phase 1 (acquisition) rats were trained to press a lever on a fixed‐ratio (FR1) schedule; each press provided one reinforcer (dustless precision pellets, 45‐mg rodent purified diet milk flavour). Concluded when adult and aged rats completed 60 trials and adolescents 30 trials or after 30 min/day. In Phase 2 (magnitude discrimination), one lever delivered five reinforcers per press, whereas the other delivered one; lever identity was randomized. Acquisition was defined as ≥ 80% correct responses overall and ≥ 85% on the five‐pellet lever for three consecutive days.

#### 2.1.3 Test

Delays appear to the high‐reward lever increasing progressively (2, 4, 8, 16, 32 and 64 s) per session. The single‐pellet lever continued delivering reward immediately. Preference was calculated as the percentage of responses to obtain the larger reinforcer based on the total responses generated in each delay session. The dependent variable was the percentage of responses on the high‐reward lever [(number of high‐reward presses ÷ total presses) × 100].

Previously, the average amount of standard food consumed in 24 h was determined: Adolescent rats consume 5 g, and adult and old rats consume 15 g. During the experiment, this amount was reduced by the number of pellets consumed to maintain motivation to seek the reinforcer (as reported by [19, 20]).

#### 2.1.4 Impulsivity Index

The *k* value was calculated, using the formula *V* = *A* / (1 + *kD*) [23], where *V* is the percent of choices for a large reward after a delay of D in seconds. *k* value describes how rapidly *V* declines with increasing delay. A higher *k* value indicates greater discounting of delayed rewards or greater impulsivity.

### 2.2 Conditioned Place Preference (CPP)

The conditioned place preference (CPP) task reflects the motivational value of a stimulus by measuring the time spent in an environment previously paired with rewarding experiences, such as palatable food, social interaction or drug effects. Increased time spent in the drug‐paired compartment is interpreted as an indicator of the rewarding or reinforcing properties of the substance [24, 25]. In independent groups for adolescents (*n* = 10), adults (*n* = 10) and aged (*n* = 9), we used 10% alcohol to consume voluntarily, calling it alcohol place preference conditioning (ACPP).

Alcohol intake was determined by weighing the ethanol bottles immediately before and after each conditioning session. Given the density of the ethanol solution (assumed to be 1 g/mL), this value was then normalized to each animal's body weight and expressed as (g ethanol per 100 g body weight).

#### 2.2.1 Apparatus

Consists of two compartments (20 × 30 × 30 cm) and a hallway (10 × 30 × 30 cm) with distinct visual cues, horizontal or diagonal black stripes. Each compartment has a fixed, nondrip drinking bottle.

#### 2.2.2 Preconditioning

On Day 1, the rat was placed in the box hallway with the compartment doors closed. After 60 s, the doors were opened, allowing free access to the compartments for 15 min. Sessions were recorded and analysed offline, registering the time in seconds the animal spent in each of the compartments, where the least preferred compartment was the one in which the animal spent the least amount of time.

#### 2.2.3 Conditioning

During the conditioning phase, the rats were given a 10% (*v*/*v*) alcohol bottle in the less preferred compartment during the preconditioning phase and a water bottle in the preferred compartment, alternating the confinement to each compartment between sessions. In the other compartment, a water bottle was provided. This phase lasted for 10 days, with 5 days of access to water and 5 days of access to alcohol alternating, each lasting 30 min. The first day of access to water or alcohol was randomized and counterbalanced. The rats were deprived of water 24 h before each day of the conditioning phase. Bottles were weighed before and after each conditioning session to record the liquid consumed.

#### 2.2.4 Test

During the test, the subjects were given 15 min to explore the entire conditioning chamber without any liquid provided. The session was recorded, and the time spent in each compartment was quantified offline.

Data are expressed as mean ± SEM of the time subjects spent in each compartment and ACPP Score. The ACPP Score is defined as the difference in time spent in the alcohol‐paired compartment during the CPP test minus the time spent in the same compartment during the preconditioning session.

After each session, the subjects had access to water for 1 h, and food was provided ad libitum.

### 2.3 Immunofluorescence

We use it to explore the CB1R, NeuN and nuclear staining 4′,6‐diamidino‐2‐phenylindole (DAPI). Additionally, CD68 (macrophage and monocyte marker) was used to endorse neuronal age [26]; see the Supporting Information for methods and results. All subjects used in the DDT study were included.

#### 2.3.1 Tissue Collection

Subjects were anaesthetized with sodium pentobarbital (126 mg/kg, i.p.) to undergo transcardial perfusion with phosphate‐buffered saline (PBS), followed by a 4% paraformaldehyde (PFA) solution. The cerebellum was removed and preserved in 4% PFA and, 24 h later, immersed in a 30% sucrose solution. Cerebellar slices, 35‐μm thick, were obtained using a Leica CM1510‐3 cryostat. The areas of interest were selected based on the rat brain atlas [27], including Crus II and the anterior interpositus nucleus from sagittal cerebellum sections.

#### 2.3.2 Immunostaining

Blocking and permeabilization were carried out using a solution of 1% BSA + 0.2% Triton X‐100 diluted in PBS, for 2 h at room temperature. After washing, the primary antibody was added. The CB1R was used at 1:200 (Abcam polyclonal Rabbit ab23703), NeuN at 1:200 (Millipore monoclonal mouse MAB377) and CD68 at 1:200 (Thermo Fisher monoclonal mouse 14‐0681). After washing, slices were incubated with Alexa Fluor 488 goat antirabbit (ab15007) and Alexa Fluor 594 goat antimouse (ab150116) secondaries (1:200) for 2 h at room temperature, mounted and coverslipped with Vectashield + DAPI.

#### 2.3.3 Microscopy and Quantification

The images were acquired with a microscope, OLYMPUS, and analysed using Image‐Pro Plus Ver. 5.1 Software, and the area occupied by the fluorescent label of the CB1R was quantified.

All images were analysed with the Fiji package (https://imagej.net/software/fiji/). A macro was created in Fiji to automate signal analysis for each channel, quantifying the percentage of area with signal relative to the optical field using an appropriate threshold according to established protocols by [20].

### 2.4 Statistical Analysis

For all experiments, values were expressed, as mean ± SEM normality tests (Shapiro–Wilk) and equality of variance tests (Levene's) were performed for all data. Data obtained from DTT and ACPP were analysed using a two‐way mixed analysis of variance (ANOVA). A sphericity test (Mauchly test) was performed for repeated measures ANOVA. A Greenhouse–Geisser correction was used when sphericity was violated. Data obtained from *k* value, the area under the curve (AUC), ACPP score, alcohol consumption and the percentages of fluorescent areas for CB1R and NeuN were analysed using one‐way independent group ANOVA. When homogeneity was violated, a Welch‐corrected ANOVA was applied. Post hoc comparisons for significant variables were conducted using Tukey's Honest Significant Difference (HSD) test.

A Pearson product–moment correlation coefficient or a Spearman's rank correlation coefficient when data are not normally distributed was computed to assess the relationship between alcohol consumption intake versus ACPP score, and CB1R fluorescent area percentages versus *k* values.

For all experiments *p* < 0.05 was considered statistically significant. Statistical analyses were conducted using the open‐source software jamovi ([28], Version 2.5).

## 3 Results

### 3.1 Delay Discounting Task (DDT)

#### 3.1.1 Training

**FIGURE 2:**
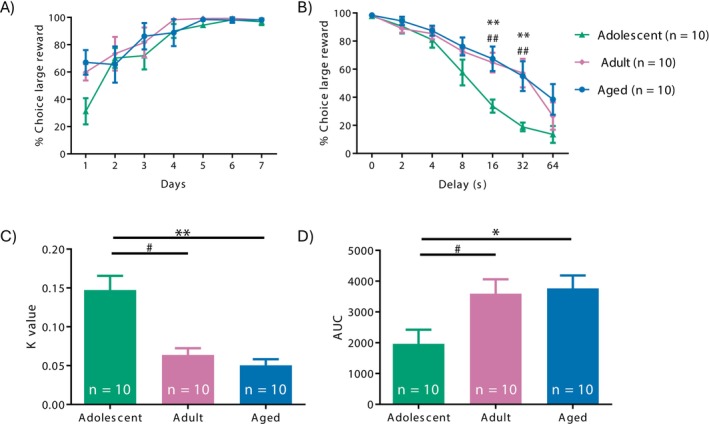
Delay discounting task (DTT). (A) Mean percentages of choices of the larger reinforcer during the training phase. (B) Mean percentages of choices of the larger reinforcer as a function of the delay to reinforcement (0, 2, 4, 8, 16, 32 and 64). **p < 0.01 adolescent versus aged rats; ^##^ *p* < 0.01 adolescent versus adult rats. (C) Discounting degree parameter (*k*). (D) Area under the curve (AUC) of the delay discounting task curve. *p < 0.05 adolescent versus aged rats, **p < 0.01 adolescent versus aged rats and ^#^ *p* < 0.05 adolescent versus adult rats.

The ANOVA indicates that the learning curve shows no statistically significant differences among adolescent, adult and aged rats (age × day interaction: *F*[4.36, 58.82] = 1.262, *p* = 0.294, *η*_*p*_2 = 0.086) (Figure 2A), indicating that all three groups acquired at a similar rate.

#### 3.1.2 Performance

A significant main effect of age (*F*_[2, 27]_ = 5.292, *p* = 0.012, *η*_*p*_^2^ = 0.282) and delay (*F*_[3.23, 87.24]_ = 74.053, *p* < 0.001, *η*_*p*_^2^ = 0.733) was found. Also, a significant age x delay interaction was found (*F*_[6.46, 87.24]_ = 2.71, *p* = 0.016, *η*_*p*_^2^ = 0.167). Post hoc comparisons revealed that adolescent rats selected the smaller immediate reward significantly more often than adults at the 16‐s delay, as indicated by the percentage of smaller‐reward choices (33.74% ± 4.73% in adolescents vs. 64.15% ± 7.00% in adults; *p* = 0.004) and 32‐s delays (18.872 ± 3.064 vs. 57.175 ± 10.161, *p* = 0.004). At the 16‐s delay, adolescent rats selected the smaller immediate reward significantly more often than aged rats, indicating a greater preference for immediacy that may reflect increased impulsive choice (33.744 ± 4.728 vs. 67.872 ± 8.763, *p* = 0.002) and 32‐s delays (18.872 ± 3.064 vs. 54.219 ± 10.612, *p* = 0.007) (Figure 2B). No significant group differences were observed at 0‐, 2‐, 4‐ or 8‐s delays.

The post hoc analysis revealed that adolescent rats made significantly more choices of the smaller, immediate reward at longer delays (16 and 32 s). This pattern is the inverse of what is shown in Figure 2B, where adolescent rats had fewer choices of the larger, delayed reward compared to adults and aged rats. To ensure clarity, we explicitly describe the data in terms of the dependent variable first, followed by its inverse when relevant.

#### 3.1.3 Impulsivity Index (*k* Value) and AUC

The ANOVA for the *k* value demonstrated a significant main effect of age (*F*_[2, 15.97]_ = 3.788, *p* = 0.045, *η*_*p*_^2^ = 0.322), with adolescent rats displaying higher impulsivity scores than adults (0.147 ± 0.018 vs. 0.063 ± 0.018, *p* = 0.022, *d* = 1.275) and aged rats (0.147 ± 0.018 vs. 0.051 ± 0.006, *p* = 0.007, *d* = 1.479) (Figure 2C). Similarly, the AUC analysis revealed a significant effect of Age (*F*_[2, 12]_ = 4.6, *p* = 0.033, *η* ^2^ = 0.353), with adolescent rats showing lower AUC values (1942 ± 459.541) compared to adults (3535 ± 464.801, *p* = 0.049, *d* = −1.371) and aged rats (3749 ± 420.590, *p* = 0.028, *d* = −1.517) (Figure 2D).

### 3.2 Alcohol‐Conditioned Place Preference (ACPP)

#### 3.2.1 Time Spent in the Alcohol‐Paired Compartment

**FIGURE 3:**
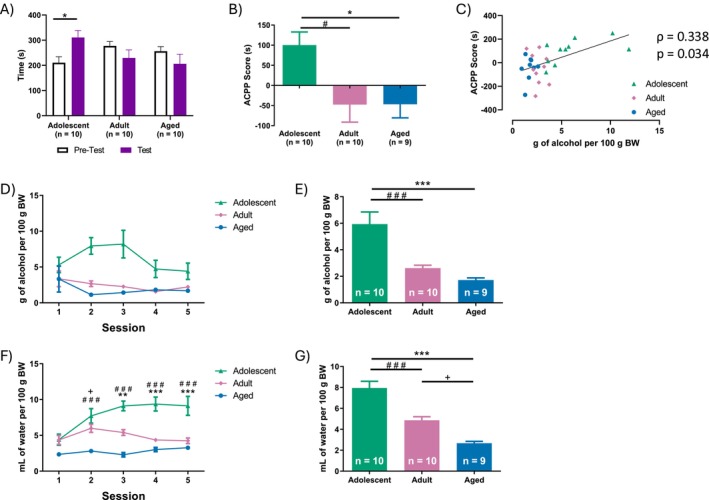
Alcohol conditioned place preference (ACPP). (A) Time spent in the alcohol‐paired compartment during the pretest and test phases. *p < 0.05. (B) ACPP score (time spent in the alcohol‐paired compartment during the test phase; time spent in the same compartment during the pretest phase). *p < 0.05 adolescent versus aged rats, ^#^ *p* < 0.05 adolescent versus adult rats. (C) Correlation between ACPP score and voluntary alcohol consumption during conditioning sessions. (D) Voluntary alcohol consumption per conditioning sessions. (E) Average of voluntary alcohol consumption during conditioning sessions. (F) Water consumption per conditioning sessions. **p < 0.05 adolescent versus aged rats, ***p < 0.001 adolescent versus aged rats, ^###^ *p* < 0.001 adolescent versus adult rats and ^+^ *p* < 0.05 adult versus aged rats.

ANOVA indicated a significant age x phase interaction (*F*_[2, 27]_ = 3.629, *p* = 0.04, *η*_*p*_^2^ = 0.212). Post hoc analysis revealed that adolescent rats spent significantly more time in the alcohol‐paired compartment (210.47 s pretest vs. 310.87 s test; *p* = 0.020) (Figure 3A). No significant differences were found in the time spent in the alcohol‐paired compartment in adult (277.24 s ± 17.492 pretest vs. 229.55 ± 31.857; *p* = 0.251) and aged rats (237.89 s ± 26.680 pretest vs. 224.58 ± 33.172; *p* = 0.746).

#### 3.2.2 ACPP Score

The score differed significantly across age groups (*F*_[2, 27]_ = 3.629, *p* = 0.04, *η*_*p*_^2^ = 0.212). Post hoc tests indicated that adolescent rats had a significantly higher ACPP score compared to adults (100.4 ± 32.403 vs. −47.6 ± 43.369, *p* = 0.041, *d* = 1.151); adult and aged rats did not differ significantly (−47.6 ± 43.369 vs. −46.67 ± 33.935, *p* > 0.05) (Figure 3B).

#### 3.2.3 Correlation Between Alcohol Consumption and ACPP Score

A positive correlation was found between alcohol consumption and the ACPP score (*ρ* = 0.338, *p* = 0.034) (Figure 3C).

#### 3.2.4 Percentage of Time in the CS+ Chamber

The % of time in the CS+ chamber was not significant across age groups (*F*_[2, 26]_ = 1.961, *p* = 0.161, *η*_*p*_^2^ = 0.131).

#### 3.2.5 Alcohol Consumption During ACPP Training

Alcohol consumption per day is depicted in Figure 3D. A two‐way mixed ANOVA was performed to evaluate the effects of age and session on alcohol consumption during ACPP sessions. The analysis revealed a significant effect of age (*F*_[2, 26]_ = 16.321, *p* < 0.001, *η*_*p*_^2^ = 0.557). Also, the analysis revealed no significant effect on ACPP session (*F*_[2.3, 60.59]_ = 1.65, *p* = 0.196, *η*_*p*_^2^ = 0.06) and no significant interaction between age and ACPP session (*F*_[4.7, 60.59]_ = 1.863, *p* = 0.119, *η*_*p*_^2^ = 0.125).

The average alcohol consumption during the ACPP training is depicted in Figure 3E. ANOVA revealed a significant effect of age (*F*_[2, 26]_ = 15.146, *p* < 0.001, *η*_*p*_^2^ = 0.538). Post hoc comparisons showed that adolescent rats consumed significantly more alcohol than adults (5.933 ± 0.9161 vs. 2.614 ± 0.224, *p* < 0.001, *d* = 1.902) and aged rats (5.933 ± 0.9161 vs. 1.682 ± 0.154, *p* < 0.001, *d* = 2.437) (Figure 3B), with no significant difference between adult and aged rats in alcohol intake (2.614 ± 0.224 vs. 1.682 ± 0.154, *p* = 0.467, *d* = 0.534).

#### 3.2.6 Water Consumption During ACPP Training

Water consumption per day is depicted in Figure 3F. A two‐way mixed ANOVA was performed to evaluate the effects of age and session on water consumption during ACPP sessions. The analysis revealed significant effects of age (*F*_[2, 26]_ = 34.818, *p* < 0.001, *η*_*p*_^2^ = 0.728) and ACPP session (*F*_[2.7, 70.25]_ = 6.485, *p* < 0.001, *η*_*p*_^2^ = 0.2). It also revealed a significant interaction between age and ACPP session (*F*_[5.4, 70.25]_ = 4.981, *p* < 0.001, *η*_*p*_^2^ = 0.277). Post hoc analyses revealed that adolescents drank more water during ACPP sessions than adults in Session 3 (*p* = 0.01), Session 4 (*p* < 0.001) and Session 5 (*p* < 0.001). Additionally, adolescent rats drank more water than aged rats in Sessions 2–5 (*p* < 0.001 for all). Adult rats only drank more water than aged rats in Session 2 (*p* = 0.046).

The average alcohol consumption during the ACPP training is depicted in Figure 3G. ANOVA revealed a significant effect of age (*F*_[2, 26]_ = 34.818, *p* < 0.001, *η*_*p*_^2^ = 0.728). Post hoc comparisons showed that adolescent rats consumed significantly more water than adults (7.942 ± 2.039 vs. 4.866 ± 1.076, *p* < 0.001, *d* = 2.218) and aged rats (7.942 ± 2.039 vs. 2.67 ± 0.518, *p* < 0.001, *d* = 3.803). Also, adult rats consume more water than aged rats (4.866 ± 1.076 vs. 2.67 ± 0.518, *p* = 0.005, *d* = 1.584).

### 3.3 Immunofluorescence

#### 3.3.1 CB1R Expression in CRUS II and Interposed Nucleus

**FIGURE 4:**
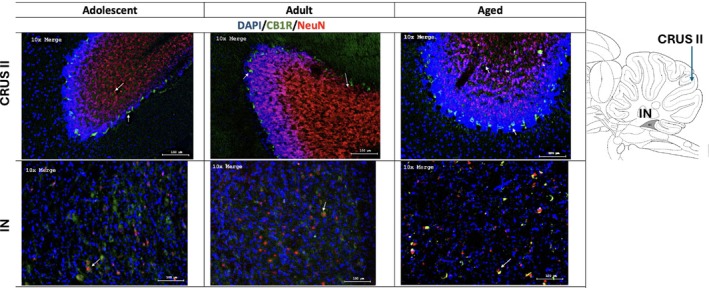
Representative images of cerebellar expression of DAPI (blue), CB1R (green) and NeuN (red) in CRUS II and the interposed nucleus (IN).

**FIGURE 5:**
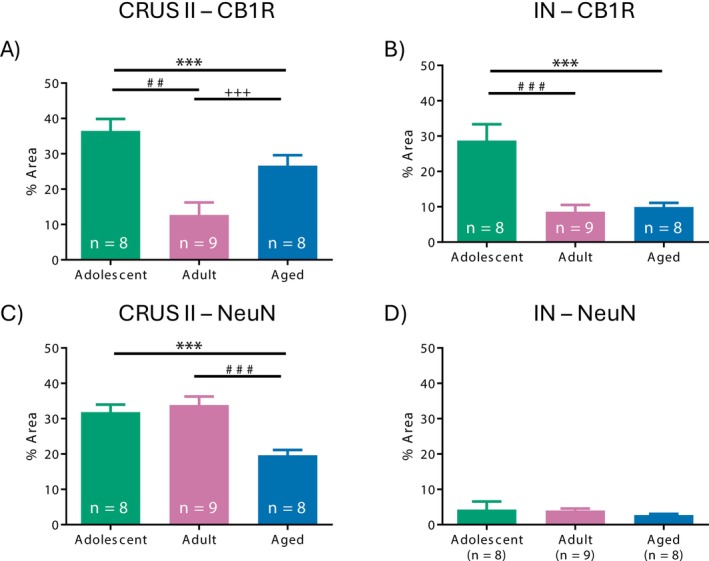
CB1R expression in CRUS II and the interposed nucleus. Percentage area covered by CB1R, NeuN in (A, C) CRUS II and (B, D) interposed nucleus (IN). ***p < 0.001 adolescent versus aged rats, ^##^ *p* < 0.01 adolescent versus adult rats, ^###^ *p* < 0.001 adolescent versus adult rats and ^+++^ *p* < 0.05 adult versus aged rats.

Analysis showed distinct CB1R expression patterns across age groups in CRUS II and the interposed nucleus (Figure 5A,B). Statistical analysis revealed a significant difference in CB1R expression in CRUS II (*F*_[2, 47]_ = 13.241, *p* < 0.001, *η*_*p*_^2^ = 0.36). Adolescent rats exhibited higher CB1R expression compared to adults (36.463 ± 2.681 vs. 8.097 ± 1.756, *p* < 0.001, *d* = 4.83) and aged rats (36.463 ± 2.681 vs. 25.692 ± 1.632, *p* < 0.001, *d* = 3.00), with a significant difference also observed between adult and aged rats (8.097 ± 1.756 vs. 25.692 ± 1.632, *p* = 0.012, *d* = 1.83). In the interposed nucleus, significant age‐related differences in CB1R expression were found (*F*_[2, 26.54]_ = 8.079, *p* = 0.002, *η*_*p*_^2^ = 0.383), with adolescent rats exhibiting higher CB1R expression compared to adults (26.488 ± 6.094 vs. 6.045 ± 1.990, *p* < 0.001, *d* = 1.663) and aged rats (26.488 ± 6.094 vs. 9.927 ± 1.499, *p* < 0.001, *d* = 1.553) (Figure 4A,B). No significant differences between adult versus old rats were found (6.045 ± 1.990 vs. 9.927 ± 1.499; *p* = 0.948).

#### 3.3.2 NeuN Expression in CRUS II and Interposed Nucleus

NeuN expression in CRUS II showed a significant effect of Age (*F*_[2, 47]_ = 13.422, *p* < 0.001, *η*_*p*_^2^ = 0.364). Post hoc comparisons revealed that adolescents exhibited significantly higher expression than aged rats (31.863 ± 2.134 vs. 19.634 ± 1.497, *p* < 0.001, *d* = 1.435). Adults also showed higher expression than aged rats (33.839 ± 2.415 vs. 19.634 ± 1.497, *p* < 0.001, *d* = 1.666) (Figure 5C). No significant difference was observed between adolescent and adult rats (31.863 ± 2.134 vs. 33.839 ± 2.415, *p* = 0.852, *d* = −0.232).

No significant age‐related differences in NeuN expression were found in the interposed nucleus (*F*_[2, 25.55]_ = 1.987, *p* = 0.158, *η*_*p*_^2^ = 0.014), indicating that neuronal density in this region remained stable across age (Figure 5D).

#### 3.3.3 Correlation Analysis

**FIGURE 6:**
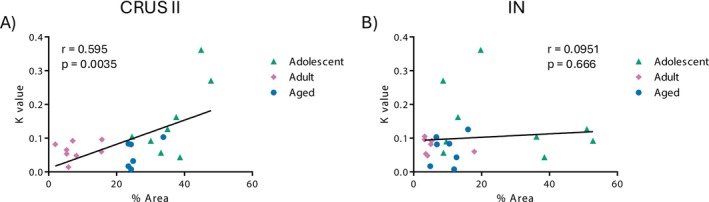
Correlation between *k* value and CB1R expression in (A) CRUS II and (B) interposed nucleus (NI).

A significant positive correlation was observed between the k value and CB1R expression in CRUS II (*r* = 0.595, *p* = 0.00350) (Figure 6A). No significant correlation was found between these variables in the anterior interposed nucleus (*r* = 0.0951, *p* = 0.666) (Figure 6B).

## 4 Discussion

This study demonstrates that adolescents exhibit a lower tolerance to delayed reinforcement compared to adult and aged rats, indicating elevated impulsivity during this developmental stage. Agreeing with previous findings [20], adolescent rats preferentially select the larger reinforcer when the delay is short (8 s or less) but shift their preference to the smaller reinforcer as the delay increases (16 s or more). In contrast, adult and aged rats maintain a preference for the larger reinforcer despite longer delays. For instance, when the larger reinforcer was delayed by 32 s, adult and aged rats chose it approximately 60% of the time, whereas adolescent rats selected it only about 18%.

These behavioural differences were paralleled by increased CB1R expression in the CRUS II and interposed nucleus of adolescent rats. Notably, only CB1R expression in the CRUS II was significantly correlated with the impulsivity index (*k* value), suggesting a region‐specific correlation to impulsivity regulation.

Previous research has implicated CB1R in adolescent impulsivity and reward sensitivity [22, 29]. For example, high CB1R expression in the nucleus accumbens has been linked to a preference for immediate rewards and increased seeking behaviour in adolescent rats [18]. Our findings highlight a novel ontogenetic pattern in which cerebellar CB1R expression coincides with heightened impulsive behaviour during adolescence. This interpretation is consistent with a growing literature showing that the cerebellum is involved in cognitive and executive functions beyond motor control [30]. Indeed, cerebellar dysfunction has been observed in disorders characterized by impulsivity and dysregulated reward processing, including attention‐deficit/hyperactivity disorder (ADHD) and substance use disorders (SUD) [14, 30].

In addition, this study indicates that aged rats perform similarly to adult rats in the DDT, exhibiting comparable tolerance to delays. Consistent findings have been reported in Fischer 344 rats, a strain characterized by accelerated aging, where young rats prefer the larger reinforcer at short delays but shift to the smaller reinforcer as delays increase, whereas aged rats maintain a preference for the larger reward [31], suggesting that the heightened impulsivity observed in adolescence is a transient developmental feature that does not persist into aging. This notion is supported by human studies, indicating that impulsivity tends to decline from adolescence to adulthood as prefrontal regulatory systems mature [32].

Adolescent rats also consumed more alcohol and developed ACPP. Adult and aged rats failed to develop ACPP, likely due to insufficient alcohol intake. A positive correlation between alcohol consumption and preference score further supports the behavioural significance of voluntary intake. These results are consistent with earlier studies showing that adolescents exhibit greater sensitivity to reward and enhanced alcohol‐seeking behaviour [20]. Unlike models using passive administration [33], our findings reflect voluntary alcohol intake, thereby increasing ecological validity.

It is important to note that although adolescent rats voluntarily consumed significantly more ethanol than adults during CPP conditioning, the amount ingested remained far below levels typically associated with pharmacological intoxication. In forced administration studies, doses of 1.5–2.0 g/kg or higher are commonly required to elicit neurobehavioural effects or blood ethanol concentrations consistent with intoxication [34, 35]. In our paradigm, the highest voluntary intake did not exceed ~0.6 g/kg, suggesting that the observed age‐related differences in place preference likely reflect increased motivational salience or reward sensitivity rather than differential intoxication.

Although adolescent‐adult differences in ethanol intake and reward sensitivity have been widely reported, our inclusion of aged rats adds a crucial dimension to the ontogenetic trajectory of alcohol‐related behaviours. The significantly lower intake observed in aged rats suggests a decline in ethanol's reinforcing properties with age, potentially linked to neurobiological or sensory changes. Moreover, by pairing ethanol exposure with a conditioned place preference paradigm, we were able to assess voluntary intake in a temporally and contextually controlled environment. This hybrid approach provides a novel and reliable means of evaluating both consumption and reward value simultaneously, particularly in populations such as aged animals where standard free‐access paradigms may underestimate motivational processes.

Although cerebellar roles in reward and addiction have attracted attention [30, 36], they remain underexplored. Our results raise the possibility that CB1R expression in CRUS II is associated with reward‐seeking behaviour, potentially through cerebellar projections to dopaminergic structures. The proposed CRUS II–VTA pathway [30] may integrate cerebellar output into mesocorticolimbic circuits. This model is further supported by human studies on cerebellar ataxia, which report increased impulsivity and emotional dysregulation [37, 38, 39].

It is worth noting that CB1R systemic activation has been associated with increased impulsive decision‐making, whereas CB1R antagonists tend to reduce impulsivity, particularly in individuals or models with elevated baseline impulsivity [40, 41]. In humans, Cnr1 polymorphism has been associated with altered prefrontal activity and impulsive traits [42]. However, the specific contribution of cerebellar CB1R—especially in CRUS II—has not been directly tested. These findings point to a region‐specific association between CB1R expression and impulsivity, in adolescence, which was not observed in the interposed nucleus.

The cerebellum, particularly its posterior regions, is increasingly recognized as a regulator of cognition and emotion [43]. Lesions in CRUS II have been associated with disinhibition and affective symptoms. Given the high CB1R density in this region [44] and its anatomical connectivity with the prefrontal cortex [45], it is plausible that CB1R activity in CRUS II modulates top‐down control processes. This hypothesis aligns with evidence indicating that adolescence is a period of dynamic remodelling in the endocannabinoid system [46].

Future studies of conditional knockdown or pharmacogenetic modulation of CB1R in CRUS II, with interposed nucleus manipulations as controls, might establish causality. By comparing adolescent, adult and aged rats, we observed that both impulsivity and cerebellar CB1R expression are highest during adolescence and decline with age. This behavioural and molecular profile suggests a potential association between cerebellar cannabinoid signalling and impulsive behaviour. The behavioural and molecular data combination suggests a novel link between cerebellar cannabinoid signalling and impulsive behaviour.

Nonetheless, the correlational nature of our data prevents us from drawing causal inferences. Furthermore, the study focused exclusively on male rats, which limits generalizability. Given documented sex differences in CB1R availability, including higher levels in males [47], future work should assess whether similar cerebellar CB1R mechanisms underlie impulsivity in females [48].

Importantly, this is the first study to report age‐related differences in CB1 receptor expression specifically within the cerebellum. Although ontogenetic and aging‐related changes in CB1R density have been documented in other brain regions, such as the hippocampus and striatum [49, 50], no previous work has systematically examined cerebellar CB1R expression across the lifespan. Our findings, therefore, provide novel evidence that CB1R expression in the cerebellum undergoes significant ontogenetic modulation, which coincides with age‐related differences in impulsive behaviour and alcohol sensitivity.

## 5 Limitations

This study has several limitations. First, its correlational design precludes causal inferences regarding the relationship between CB1R expression, impulsivity and alcohol consumption. Although our findings suggest a role for cerebellar CB1R, particularly in CRUS II, in modulating impulsive behaviour, causal mechanisms remain speculative. Future experiments involving region‐specific manipulations (e.g., pharmacological blockade, chemogenetic inhibition or conditional knockdown) are needed to determine directionality. Second, the study was limited to male rats, which restricts the generalizability of our findings. Given known sex differences in endocannabinoid signalling, including CB1R density and function, further research in females is warranted. Finally, the cross‐sectional nature of the design does not capture developmental trajectories. Longitudinal studies could help to delineate how cerebellar CB1R expression and impulsive behaviour coevolve across the lifespan.

## 6 Conclusion

This study provides new evidence of an association between CB1R expression in the cerebellum, particularly in CRUS II, and impulsivity and alcohol‐seeking behaviour in adolescent rats. These findings support the notion that the cerebellum, traditionally viewed as a motor structure, may also be involved in neural systems associated with impulse control and vulnerability to substance use. The possibility of a cerebellar‐dopaminergic interface, potentially via the CRUS II–VTA axis, warrants further investigation and may open new avenues for translational research targeting the endocannabinoid system.

## Author Contributions

JPGV and YAAR made substantial contributions to the acquisition and analysis of data for the work. OAB made significant contributions to the design, analysis, and interpretation of the work's data and revised it critically for important intellectual content. MMD conceived and designed the work and agreed to be accountable for all aspects of the work, ensuring that questions related to the accuracy and integrity of any part of the work are appropriately investigated and resolved. All authors approved the final version of the work to be published.

## Funding

This study was supported by the Dirección General de Asuntos del Personal Académico, Universidad Nacional Autónoma de México (IN202925 and IA205218).

## Ethics Statement

All authors confirm that this manuscript is original, has not been published elsewhere and is not under consideration by any other journal. We have adhered to the highest standards of research integrity and ethical conduct throughout study design, data collection, analysis and reporting.

## Conflicts of Interest

The authors declare no conflicts of interest.

## Supporting information


**Figure S1:** Representative Images of DAPI (blue), CB1R (green), and CD68 (red) expression in CRUS II and interposed nucleus (NI).
**Figure S2:** Comparison of the percentage area covered by CD68 fluorescence markers in CRUS II and interposed nucleus (IN).

## Data Availability

The datasets generated and analysed during the current study are available from the corresponding author upon reasonable request.

## References

[1] J. E. Grant and S. R. Chamberlain , “Impulsive Action and Impulsive Choice Across Substance and Behavioral Addictions: Cause or Consequence?,” Addictive Behaviors 39, no. 11 (2014): 1632–1639, 10.1016/j.addbeh.2014.04.022. 24864028

[2] J. Weafer , S. H. Mitchell , and H. de Wit , “Recent Translational Findings on Impulsivity in Relation to Drug Abuse,” Current Addiction Reports 1, no. 4 (2014): 289–300, 10.1007/s40429-014-0035-6. 25678985 PMC4323183

[3] L. Peyton , A. Oliveros , C. H. Cho , et al., “Waiting Impulsivity During Reward Seeking Increases Adult Hippocampal Neurogenesis in Mice,” Neuroscience Letters 706 (2019): 169–175, 10.1016/j.neulet.2019.05.032. 31116969

[4] R. Woods‐Gonzalez , J. T. Waddell , S. E. King , and W. R. Corbin , “Differentiating Action From Inaction: Longitudinal Relations Among Impulsive Personality Traits, Internalizing Symptoms, and Drinking Behavior,” Addictive Behaviors 154 (2024): 108019, 10.1016/j.addbeh.2024.108019. 38502991 PMC11015960

[5] A. Salguero , A. Pilatti , Y. Michelini , G. Rivarola Montejano , and R. M. Pautassi , “Impulsivity, Mental Health State and Emotion Regulation Modulate Alcohol and Marijuana Use in a Sample of Argentinean Citizens,” Alcohol 118 (2024): 37–44, 10.1016/j.alcohol.2023.11.005. 38006977

[6] K. Yan , Y. Feng , Z. Liu , W. Shi , Y. Jiang , and J. Liu , “Impulsivity Drives Adolescents to Smoke and Drink: Gender Differences in the Mediating Effects of Resilience and Depression,” Psychological Reports. Advance online publication 128 (2023): 4062–4087, 10.1177/00332941231216894. 37982432

[7] E. M. L. Wolfs , J. Klaus , and D. J. L. G. Schutter , “Cerebellar Grey Matter Volumes in Reactive Aggression and Impulsivity in Healthy Volunteers,” Cerebellum 22 (2023): 223–233, 10.1007/s12311-021-01337-5. 35247193 PMC9985584

[8] M. R. Rueda , M. I. Posner , and M. K. Rothbart , “The Development of Executive Attention: Contributions to the Emergence of Self‐Regulation,” Developmental Neuropsychology 28, no. 2 (2005): 573–594, 10.1207/s15326942dn2802_2. 16144428

[9] M. Méndez‐Díaz , D. A. Rangel Rangel , Y. A. Alvarado Ramírez , et al., “Función de la Impulsividad en el Trastorno por Consumo de Sustancias,” Psychologia 15, no. 1 (2021): 83–93.

[10] R. M. Kelly and P. L. Strick , “Cerebellar Loops With Motor Cortex and Prefrontal Cortex of a Nonhuman Primate,” Journal of Neuroscience 23, no. 23 (2003): 8432–8444, 10.1523/JNEUROSCI.23-23-08432.2003. 12968006 PMC6740694

[11] T. C. Watson , N. Becker , R. Apps , and M. W. Jones , “Back to Front: Cerebellar Connections and Interactions With the Prefrontal Cortex,” Frontiers in Systems Neuroscience 8 (2014): 4, 10.3389/fnsys.2014.00004. 24550789 PMC3912388

[12] C. Habas , N. Kamdar , D. Nguyen , et al., “Distinct Cerebellar Contributions to Intrinsic Connectivity Networks,” Journal of Neuroscience 29, no. 26 (2009): 8586–8594, 10.1523/JNEUROSCI.1868-09.2009. 19571149 PMC2742620

[13] F. Palesi , A. de Rinaldis , G. Castellazzi , et al., “Contralateral Cortico‐Ponto‐Cerebellar Pathways Reconstruction in Humans In Vivo: Implications for Reciprocal Cerebro‐Cerebellar Structural Connectivity in Motor and Non‐Motor Areas,” Scientific Reports 7 (2017): 12841, 10.1038/s41598-017-13079-8. 28993670 PMC5634467

[14] T. X. Chen , C.‐Y. R. Lin , M. A. Aumann , et al., “Impulsivity Trait Profiles in Patients With Cerebellar Ataxia and Parkinson Disease,” Neurology 99 (2022): e176–e186, 10.1212/WNL.0000000000200349. 35428731 PMC9280994

[15] A. Tessier , C. Cosin , W. Mayo , M. Pfeuty , D. Misdrahi , and I. Sibon , “Impulsive Aggressive Obsessions Following Cerebellar Strokes: A Case Study,” Journal of Neurology 262, no. 8 (2015): 1775–1776, 10.1007/s00415-015-7804-6. 26048690

[16] M. E. Sim , I. K. Lyoo , C. C. Streeter , et al., “Cerebellar Gray Matter Volume Correlates With Duration of Cocaine Use in Cocaine‐Dependent Subjects,” Neuropsychopharmacology 32, no. 10 (2007): 2229–2237, 10.1038/sj.npp.1301346. 17299505

[17] E. V. Sullivan , T. Brumback , S. F. Tapert , et al., “Disturbed Cerebellar Growth Trajectories in Adolescents Who Initiate Alcohol Drinking,” Biological Psychiatry 87, no. 7 (2020): 632–644, 10.1016/j.biopsych.2019.11.014. 31653477 PMC7061065

[18] Z. Cai , P. Wang , B. Liu , et al., “To Explore the Mechanism of Tobacco Addiction Using Structural and Functional MRI: A Preliminary Study of the Role of the Cerebellum‐Striatum Circuit,” Brain Imaging and Behavior 16, no. 2 (2021): 834–842, 10.1007/s11682-021-00546-0. 34606038

[19] O. Amancio‐Belmont , A. Romano‐López , A. E. Ruiz‐Contreras , M. Méndez‐Díaz , and O. Prospéro‐García , “From Adolescent to Elder Rats: Motivation for Palatable Food and Cannabinoids Receptors,” Developmental Neurobiology 77, no. 8 (2017): 917–927, 10.1002/dneu.22472. 27935269

[20] B. Romero‐Torres , Y. Alvarado‐Ramírez , S. Duran‐Alonzo , et al., “A Potential Role of Hippocampus on Impulsivity and Alcohol Consumption Through CB1R,” Pharmacology Biochemistry and Behavior 225 (2023): 173558, 10.1016/j.pbb.2023.173558. 37088449

[21] O. Amancio‐Belmont , A. L. Becerril Meléndez , A. E. Ruiz‐Contreras , M. Méndez‐Díaz , and O. Prospéro‐García , “Opposed Cannabinoid 1 Receptor (CB1R) Expression in the Prefrontal Cortex vs. Nucleus Accumbens Is Associated With Alcohol Consumption in Male Rats,” Brain Research 1725 (2019): 146485, 10.1016/j.brainres.2019.146485. 31568767

[22] M. Schneider , F. Kasanetz , D. L. Lynch , et al., “Enhanced Functional Activity of the Cannabinoid Type‐1 Receptor Mediates Adolescent Behavior,” Journal of Neuroscience 35, no. 41 (2015): 13975–13988, 10.1523/JNEUROSCI.1937-15.2015. 26468198 PMC4604232

[23] J. S. Stein , J. W. Pinkston , A. T. Brewer , M. T. Francisco , and G. J. Madden , “Delay Discounting in Lewis and Fischer 344 Rats: Steady‐State and Rapid‐Determination Adjusting‐Amount Procedures,” Journal of the Experimental Analysis of Behavior 97, no. 3 (2013): 305–321, 10.1901/jeab.2012.97-305. PMC3372954 22693360

[24] M. T. Bardo and R. A. Bevins , “Conditioned Place Preference: What Does It Add to Our Preclinical Understanding of Drug Reward?,” Psychopharmacology 153, no. 1 (2000): 31–43, 10.1007/s002130000569. 11255927

[25] T. M. Tzschentke , “Measuring Reward With the Conditioned Place Preference (CPP) Paradigm: Update of the Last Decade,” Addiction Biology 12, no. 3–4 (2007 Sep): 227–462, 10.1111/j.1369-1600.2007.00070.x. 17678505

[26] M. Farso , C. Ménard , J. Colby‐Milley , and R. Quirion , “The Immune Marker CD68 Correlates With Cognitive Impairment in Normally Aged Rats,” Neurobiology of Aging 34, no. 8 (2013): 1971–1976, 10.1016/j.neurobiolaging.2013.02.008. 23523271

[27] J. H. Paxinos and S. J. Watson , “The Rat Brain in Stereotaxic Coordinates (2nd ed.),” Trends in Neurosciences 10, no. 10 (1987): 439, 10.1016/0166-2236(87)90017-8.

[28] The jamovi Project , “jamovi (Version 2.5),” (2024), https://www.jamovi.org.

[29] M. Méndez‐Díaz , “The Hippocampus and Impulsivity: Linking in Cannabinoid Receptor 1,” in Handbook of the Biology and Pathology of Mental Disorders, (Springer, 2025), 10.1007/978-3-031-32035-4_43-1.

[30] M. Miquel , S. M. Nicola , I. Gil‐Miravet , J. Guarque‐Chabrera , and A. Sanchez‐Hernandez , “A Working Hypothesis for the Role of the Cerebellum in Impulsivity and Compulsivity,” Frontiers in Behavioral Neuroscience 13 (2019): 99, 10.3389/fnbeh.2019.00099. 31133834 PMC6513968

[31] N. W. Simon , C. L. LaSarge , K. S. Montgomery , et al., “Good Things Come to Those Who Wait: Attenuated Discounting of Delayed Rewards in Aged Fischer 344 Rats,” Neurobiology of Aging 31, no. 5 (2010): 853–862, 10.1016/j.neurobiolaging.2008.06.004. 18657883 PMC2866647

[32] S. Hamidullah , H. H. A. Thorpe , J. A. Frie , R. D. McCurdy , and J. Y. Khokhar , “Adolescent Substance Use and the Brain: Behavioral, Cognitive and Neuroimaging Correlates,” Frontiers in Human Neuroscience 14 (2020): 298, 10.3389/fnhum.2020.00298. 32848673 PMC7418456

[33] M. Morales , E. I. Varlinskaya , and L. P. Spear , “Evidence for Conditioned Place Preference to a Moderate Dose of Ethanol in Adult Male Sprague–Dawley Rats,” Alcohol 46, no. 7 (2012): 643–648, 10.1016/j.alcohol.2012.06.001. 22784435 PMC3455131

[34] J. C. Crabbe , P. Metten , J. S. Rhodes , et al., “A Line of Mice Selected for High Blood Ethanol Concentrations Shows Drinking in the Dark to Intoxication,” Biological Psychiatry 65, no. 8 (2009): 662–670, 10.1016/j.biopsych.2008.11.002. 19095222 PMC3330756

[35] E. Majchrowicz , “Induction of Physical Dependence Upon Ethanol and the Associated Behavioral Changes in Rats,” Psychopharmacologia 43, no. 3 (1975): 245–254, 10.1007/BF00429258. 1237914

[36] I. Gil‐Miravet , J. Guarque‐Chabrera , M. Carbo‐Gas , F. Olucha‐Bordonau , and M. Miquel , “The Role of the Cerebellum in Drug‐Cue Associative Memory: Functional Interactions With the Medial Prefrontal Cortex,” European Journal of Neuroscience 50, no. 3 (2019): 2613–2622, 10.1111/ejn.14187. 30280439

[37] N. Amokrane , C. R. Lin , N. A. Desai , and S. H. Kuo , “The Impact of Compulsivity and Impulsivity in Cerebellar Ataxia: A Case Series,” Tremor and Other Hyperkinetic Movements 10 (2020): 43, 10.5334/tohm.550. 33133767 PMC7583703

[38] S. Carmona , O. Vilarroya , A. Bielsa , et al., “Global and Regional Gray Matter Reductions in ADHD: A Voxel‐Based Morphometric Study,” Neuroscience Letters 389, no. 2 (2005): 88–93, 10.1016/j.neulet.2005.07.020. 16129560

[39] H. Kirchner , O. Kremmyda , K. Hüfner , et al., “Clinical, Electrophysiological, and MRI Findings in Patients With Cerebellar Ataxia and a Bilaterally Pathological Head‐Impulse Test,” Annals of the New York Academy of Sciences 1233 (2011): 127–138, 10.1111/j.1749-6632.2011.06175.x. 21950985

[40] T. Pattij , J. Wiskerke , and A. N. Schoffelmeer , “Cannabinoid Modulation of Executive Functions,” European Journal of Pharmacology 585, no. 2–3 (2008): 458–463, 10.1016/j.ejphar.2008.02.099. 18423599

[41] J. Wiskerke , T. Pattij , A. N. Schoffelmeer , and T. J. de Vries , “The Role of CB1 Receptors in Psychostimulant Addiction,” Addiction Biology 13, no. 2 (2008): 225–238, 10.1111/j.1369-1600.2008.00109.x. 18482432

[42] J. Zeng , X. Zhao , H. Qin , X. Hou , and Q. Zhang , “Do Genes Play a Role in the Decoy Effect?,” Frontiers in Psychology 11 (2020): 523299, 10.3389/fpsyg.2020.523299. 33192763 PMC7606847

[43] J. D. Schmahmann and J. C. Sherman , “The Cerebellar Cognitive Affective Syndrome,” Brain 121, no. 4 (1998): 561–579, 10.1093/brain/121.4.561. 9577385

[44] M. Herkenham , A. B. Lynn , M. D. Little , et al., “Cannabinoid Receptor Localization in Brain,” Proceedings of the National Academy of Sciences 87, no. 5 (1990): 1932–1936, 10.1073/pnas.87.5.1932. PMC53598 2308954

[45] R. L. Buckner , F. M. Krienen , A. Castellanos , J. C. Diaz , and B. T. Yeo , “The Organization of the Human Cerebellum Estimated by Intrinsic Functional Connectivity,” Journal of Neurophysiology 106, no. 5 (2011): 2322–2345, 10.1152/jn.00339.2011. 21795627 PMC3214121

[46] M. Verdurand , V. Nguyen , D. Stark , et al., “Comparison of Cannabinoid CB1 Receptor Binding in Adolescent and Adult Rats: A Positron Emission Tomography Study Using [F]MK‐9470,” International Journal of Molecular Imaging 2011 (2011): 548123, 10.1155/2011/548123. 22187642 PMC3236487

[47] H. Laurikainen , L. Tuominen , M. Tikka , et al., “Sex Difference in Brain CB1 Receptor Availability in Man,” NeuroImage 184 (2019): 834–842, 10.1016/j.neuroimage.2018.10.013. 30296558

[48] A. Flores‐Bonilla and H. N. Richardson , “Sex Differences in the Neurobiology of Alcohol Use Disorder,” Alcohol Research: Current Reviews 40, no. 2 (2020): 4, 10.35946/arcr.v40.2.04. PMC7532022 33042719

[49] A. Bilkei‐Gorzo , “The Endocannabinoid System in Normal and Pathological Brain Ageing,” Philosophical Transactions of the Royal Society, B: Biological Sciences 367, no. 1607 (2012): 3326–3341, 10.1098/rstb.2011.0388. PMC3481530 23108550

[50] J. Romero , E. Garcia‐Palomero , F. Berrendero , et al., “Atypical Location of Cannabinoid Receptors in White Matter Areas During Rat Brain Development,” Synapse 26, no. 3 (1997): 317–323, 10.1002/(SICI)1098-2396(199707)26:3<317::AID-SYN12>3.0.CO;2-S. 9183820

