# Supplementary material for: Age‐Dependent Differences in Cerebellar CB1 Receptor Expression and Its Association With Impulsivity and Alcohol Intake in Rats
Source: Addict Biol. 2025 Nov 29;30(12):e70107. doi: 10.1111/adb.70107 (PMC12664822; doi:10.1111/adb.70107)
Supplement: Supplementary file 1 — Figure S1: Representative Images of DAPI (blue), CB1R (green), and CD68 (red) expression in CRUS II and interposed nucleus (NI). Figure S2: Comparison of the percentage area covered by CD68 fluorescence markers in CRUS II and interposed nucleus (IN). [file ADB-30-e70107-s001.pdf]

Supplementary

CD68 immunofluorescence method and results

Immediately after the TDD test, the rats were transcardially perfused with phosphate-buffered saline (PBS) followed by 4% paraformaldehyde (PFA), pH 7.4. Subsequently, the tissue was dehydrated with 30% sucrose and then sectioned into 35 µm coronal slices using a freezing microtome (Leica, CM 1510-3). Immunofluorescence was performed using the free-floating technique on coronal sections containing CRUS II and interposed nucleus (n= 7 adolescent, n=8 adults and n=6 aged). The sections were washed with PBS, followed by blocking and permeabilization with a solution of 1% BSA + 0.2% Triton X-100 diluted in PBS for 2 hours at room temperature. After complete blocking, the sections were incubated with primary antibodies. We used a rabbit polyclonal anti-CB1R antibody (ab23703, 1:200) and a mouse monoclonal anti-CD68 antibody (Thermo Fisher 14-0681, 1:200). All primary antibodies were incubated for 24 hours at 4°C. After three washes with PBS, the sections were incubated with the corresponding secondary antibodies: Alexa Fluor 488 anti-rabbit (ab15007, 1:200) and Alexa Fluor 594 anti-mouse (ab150116, 1:200) at room temperature for 2 hours. Finally, the sections were mounted on pre-gelatinized slides. Sections were observed and analyzed as described in the immunofluorescence method of the main article.

CD68 expression in the CRUS II show significant differences $F(2,19) = 21.516$, $p < 0.001$. Post hoc analyze show differences between adolescent vs. aged rats (M ± SEM 0.811 ± 0.171 vs. 4.856 ± 0.566, $p < 0.001$) and adult vs. aged rats (M ± SEM 1.854 ± 0.463 vs. 4.856 ± 0.566, $p < 0.001$). In the interposed nucleus, there were significant differences ($F(2,26.7) = 13.1$, $p < 0.001$). Post hoc analysis revealed adolescents show less expression of CD58 than aged rats (M ± SEM 0.895 ± 0.503 vs. 5.207 ± 0.830, $p < 0.001$) and between adult and aged rats (M ± SEM 0.825 ± 0.218 vs. 5.207 ± 0.830, $p < 0.001$).

We found a higher expression of CD68 in aged rats, compared to adolescent and adult rats. These results are consistent with the literature, where it has been shown that CD68 is more expressed in aged subjects. In aging, microglia often present a more pro-inflammatory and dysfunctional state, contributing to chronic neuroinflammation and neuronal deterioration. This change is associated with an increase in the expression of markers such as CD68 and the production of pro-inflammatory cytokines. In young individuals, microglia remain more homeostatic and active in a more controlled manner in response to injury or infection (Norden and Godbout, 2013; Luo and Chen, 2012).

Figure S1

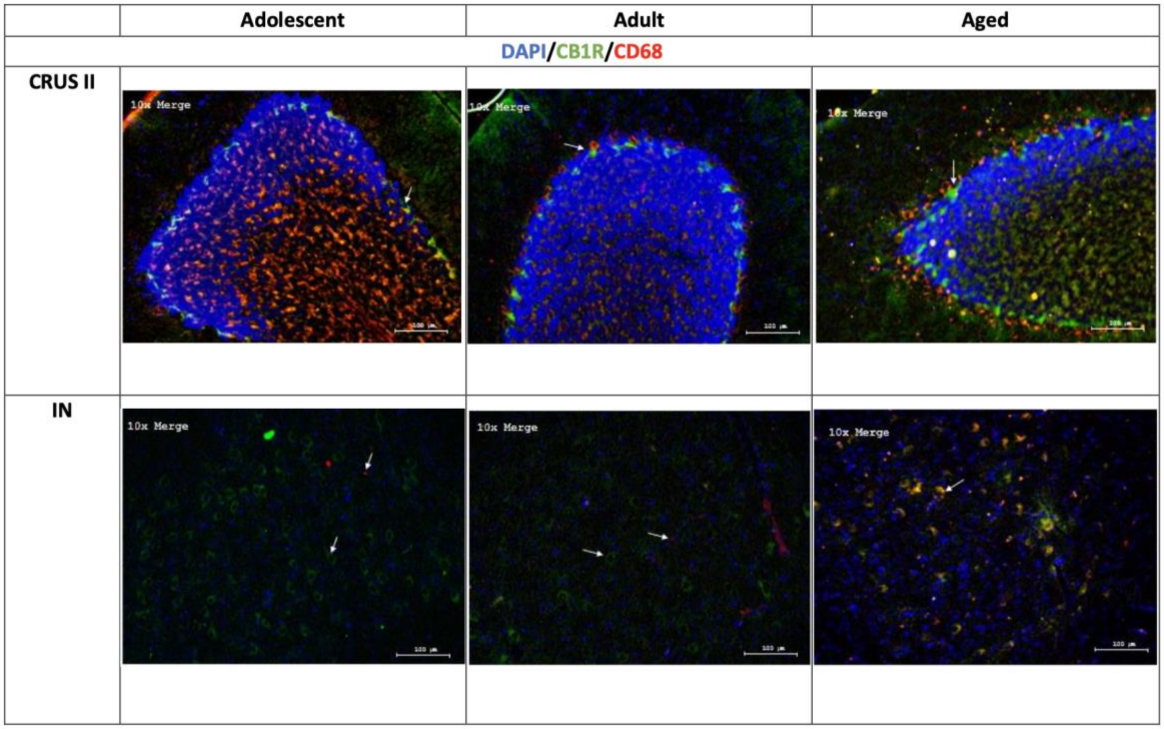

Figure 1. Representative Images of DAPI (blue), CB1R (green), and CD68 (red) expression in CRUS II and interposed nucleus (NI).

Figure S1

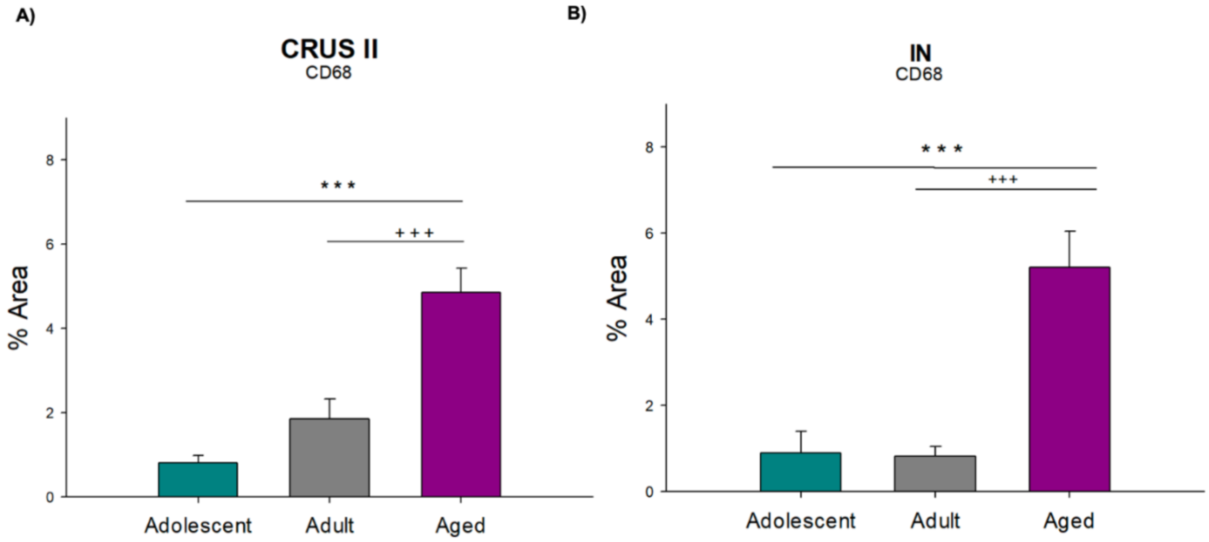

Figure 2. Comparison of the percentage area covered by CD68 fluorescence markers in CRUS II and interposed nucleus (IN).
